# A natural in situ fabrication method of functional bacterial cellulose using a microorganism

**DOI:** 10.1038/s41467-018-07879-3

**Published:** 2019-01-25

**Authors:** Minghong Gao, Juan Li, Zixian Bao, Moudong Hu, Rui Nian, Dexin Feng, Dong An, Xing Li, Mo Xian, Haibo Zhang

**Affiliations:** 1 CAS Key Laboratory of Biobased Materials, Qingdao Institute of Bioenergy and Bioprocess Technology, Chinese Academy of Sciences, No. 189 Songling Road, Qingdao, 266101 China; 2 College of Chemistry and Chemical Engineering, Ocean University of China, No. 238 Songling Road, Qingdao, 266003 China

## Abstract

The functionalization methods of materials based on bacterial cellulose (BC) mainly focus on the chemical modification or physical coating of fermentation products, which may cause several problems, such as environment pollution, low reaction efficiency and easy loss of functional moieties during application. Here, we develop a modification method utilizing the in situ microbial fermentation method combined with 6-carboxyfluorescein-modified glucose (6CF-Glc) as a substrate using *Komagataeibacter sucrofermentans* to produce functional BC with a nonnatural characteristic fluorescence. Our results indicate that the microbial synthesis method is more efficient, controllable and environmentally friendly than traditional modification methods. Therefore, this work confirms that BC can be functionalized by using a microbial synthesis system with functionalized glucose, which provides insights not only for the functionalization of BC but also for the in situ synthesis of other functional materials through microbial synthetic systems.

## Abstract (web summary)

Bacterial cellulose (BC) is a ubiquitous biomaterial, but functionalization relies on modification processes which have low reaction efficiency and can cause pollution. Here the authors develop an environmental friendly in-situ fermentation method using fluorescein labelled glucose to produce fluorescent BC.

## Introduction

Functional materials constitute an extremely active and breakthrough research area in which all aspects of materials science are involved, including chemistry, physics, engineering, biology, and nanotechnology. Different forms of functional materials have been developed for various purposes, such as environmental monitoring, food industry, ion battery fabrication, and clinical therapy^1–6^. Of them, functional materials based on bacterial cellulose (BC) have attracted increasing attention because of its excellent physical and chemical properties, including high safety, low cost, high flexibility and adaptability, hydrophilicity, transparency, excellent biocompatibility and biodegradability^7,8^. BC is a porous network-like nanoscale biopolymer produced by the fermentation of microorganisms, which consists of chains of β-D-glucose linked by β-1,4-glycosidic bonds. During the fermentation, glucose is metabolized by microorganisms and forms into linear β-1,4-glucan chains. These cellulose linear chains are secreted extracellularly and crystallized to form cellulose monofilaments. A certain number of monofilaments aggregate to form filamentous fibers (like ribbons), which further form a three-dimensional and gelatinous structure on the surface of a liquid medium^9–11^. The bacterial strains that produce BC include *Acetobacter, Rhizobium, Xanthococcus, Pseudomonas, Azotobacter, Aerobacter*, and *Alcaligenes*. Among them, *Komagataeibacter xylinum* (formerly *Gluconacetobacter xylinum*) is the most commonly used microorganism to synthesize BC due to its high yield. In our work, *Komagataeibacter sucrofermentans* (*Komagataeibacter xylinum* subsp. *sucrofermentans*, *K. sucrofermentans*) was used for BC production due to its ability to synthesize cellulose stably and produce thick pellicles under static growth conditions^12^.

BC has several advantages over plant-derived cellulose, including high purity (free of lignin, hemicellulose and pectin)^13^, high crystallinity, high elasticity and conformability, low density, high specific surface area, high degree of polymerization, excellent permeability, high porosity and water content, and high mechanical strength in wet state^7,10,14–17^, ensuring the multifunction of BC. More importantly, BC is superior in terms of modification. First, it can be shaped in situ during fermentation to form different structures, such as tubes, membranes, spheres and layers of thin fibers^13^, meeting the needs of functional materials for various applications with a one-step synthesis. Second, BC has a multihydroxyl molecular structure that can be functionalized, such as by functional compound modification or polymer coating^18^. As a result, functionalized BC-based materials have emerged and are utilized in various fields, such as chemical sensing, bioimaging, UV screening, oil adsorption, fuel cells, biomedical materials, ion detection, and anti-counterfeiting labels^2,6,19–22^.

BC is often functionalized with chemical modification or physical coating based on fermentation products. Commonly used modification methods include nanoparticle coating, metal oxide modification, fluorescent substance modification and ionic liquid replacement^2,23–25^. The physical coating can provide a mild modification condition, but functional moieties potentially suffer from shedding due to weak physical interactions. The high polarity and strong intermolecular hydrogen bonding within BC lead to its poor solubility in water and standard organic solvents^26^. Therefore, although BC has a multihydroxyl molecular structure, the chemical modification of cellulose-based materials with functional groups remains plagued by a limited choice of solvents, such as N,N-dimethylacetamide/LiCl, dimethyl sulfoxide/tetrabutylammonium fluoride, N-methylmorpholine-N-oxides and ionic liquids^27,28^. However, the utilization of these toxic chemical agents has serious negative effects on the environment, simultaneously influences the green safety of products, and adds complexity to waste discharge treatment^29^, limiting its large-scale production. Moreover, chemical modification can be performed with cellulose nanocrystals or nanofibers in a heterogeneous system^2^, which may lead to a low reaction efficiency. More importantly, due to the multihydroxyl molecular structure of BC, selective modification on a specific C site of BC is difficult using chemical methods, whereas the biological synthesis method with high specificity may solve this problem. However, no functionalized modifications on BC using a microbial in situ fermentation system were reported, which may provide a potential method for biological modification of BC, with the dual goal of providing an innovative microbial modification method that circumvents the limitations of chemical and physical modification, and understanding the microbial metabolic pathways that produce functionalized products.

Recently, an innovative method for the synthesis of functional plant cellulose was reported. *Gossypium hirsutum* fertilized ovules were selected as the in vitro fabrication condition to perform in situ functionalization of cellulose fibers^30^. Using glucose as the carrier, functional molecules were introduced into plant cellulose fibers through the cotton ovule. Through the regulation of glucose and functional groups, cotton fibers with fluorescent and magnetic properties were prepared, respectively. The results indicated that the fluorescent cotton fiber is more durable and environmentally friendly than those prepared with the conventional method of compound coating modification^30^. A microbial fermentation system has several advantages over other synthesis methods, such as high efficiency, short production cycle, and ease of regulating the functionality of products. Therefore, this system is an optical research platform for synthesizing functional biobased materials^31^. However, whether this in situ modification method can be extended to other biosynthesis systems, particularly high-efficiency microbial systems, remains to be elucidated.

Here, we perform an innovative modification method for functional BC biofabrication using a microbial system. Glucose is functionally modified with 6-carboxyfluorescein (6CF) and used as a substrate to produce the functional BC by in situ fermentation with *K. sucrofermentans*. The fluorescence intensity of functional BC can be controlled by adjusting the concentration of 6CF-modified glucose (6CF-Glc) in the culture medium. Our results verify that BC could be functionally modified by a microbial synthesis system, which has superiority over chemical modification. Therefore, our approach has implications for both the modification of BC and production of other valuable functional materials via biosynthetic systems.

## Results

### Chemical preparation of functional BC

**Fig. 1:**
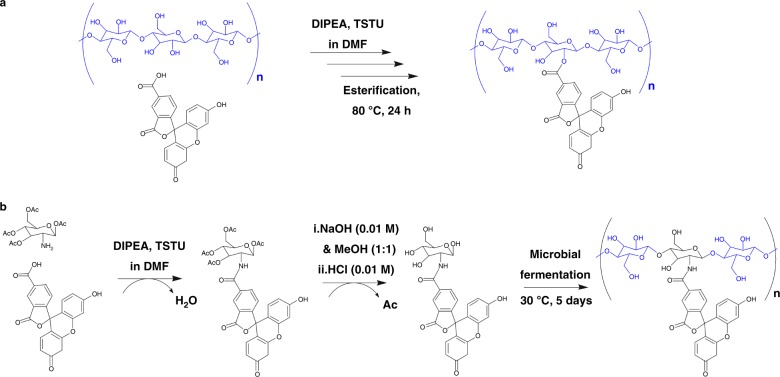
Chemical and biological preparation of functional BC. **a** Chemically modified BC (Ch-6CF/BC) was prepared by soaking neat BC in activated 6CF solutions for 30 min at room temperature under inert conditions and drying at 80 °C. **b** The 6-carboxyfluorescein-glucose (6CF-Glc) was first synthesized starting from 6-CF and 1,3,4,6-tetra-O-acetyl-2-amino-2-deoxy-β-D-glucopyranose, and it was then used as a carbon source to produce functionalized 6CF-BC through microbial fermentation

We used a green chemical method, in which cellulose-based materials were modified with fluorophores by simple immersion followed by drying above 80 °C for the preparation of functional BC^29^, and retouching of the BC with 6CF (a fluorescent dye) (Fig. 1a). Finally, we obtained the chemically modified functional BC (Ch-6CF/BC). The green fluorescence of Ch-6CF/BC was observed under ultraviolet (UV) light (365 nm) (Supplementary Figure 1). Fourier transform infrared spectroscopy with attenuated total reflectance (FT-IR ATR) analysis of the Ch-6CF/BC fibers showed no absorption peak at 1740 cm^−1^ (C=O stretching in ester), which was the same as the FT-IR ATR spectrum of BC (Supplementary Figure 2).

### Biological preparation of functional BC

Based on the biosynthesis of functional plant cellulose^30^, 6CF-Glc was used as a substrate to produce biologically modified BC (6CF-BC) by in situ fermentation of *K. sucrofermentans*. The microorganism was sensitive to the additive molecules in the medium; therefore, we first explored the selectivity of 6CF on *K. sucrofermentans*. 6CF was added directly to the fermentation medium, and *K. sucrofermentans* was cultured under standard conditions. A complete BC pellicle was produced on the surface of the culture medium (Supplementary Figure 3). The obtained BC was observed with confocal laser scanning microscopy (CLSM, emission wavelength, 488 nm), and *K. sucrofermentans* on the surface of BC exhibited fluorescence (Supplementary Figure 4), demonstrating that 6CF entered the cell body of *K. sucrofermentans* and had no clear impact on its growth and metabolism. Thus, the synthesis of fluorescent BC through the fermentation of *K. sucrofermentans* with 6CF-Glc as the carbon source is feasible. 6CF-Glc was synthesized by reacting the free amino group located at C2 of the acetyl-protected glucosamine moiety with the carboxylate from 6CF (Fig. 1b). The UV-Vis spectrum, mass spectrometry, and ^1^H NMR were further used to verify the successful synthesis of 6CF-Glc (Supplementary Figure 5).

**Fig. 2:**
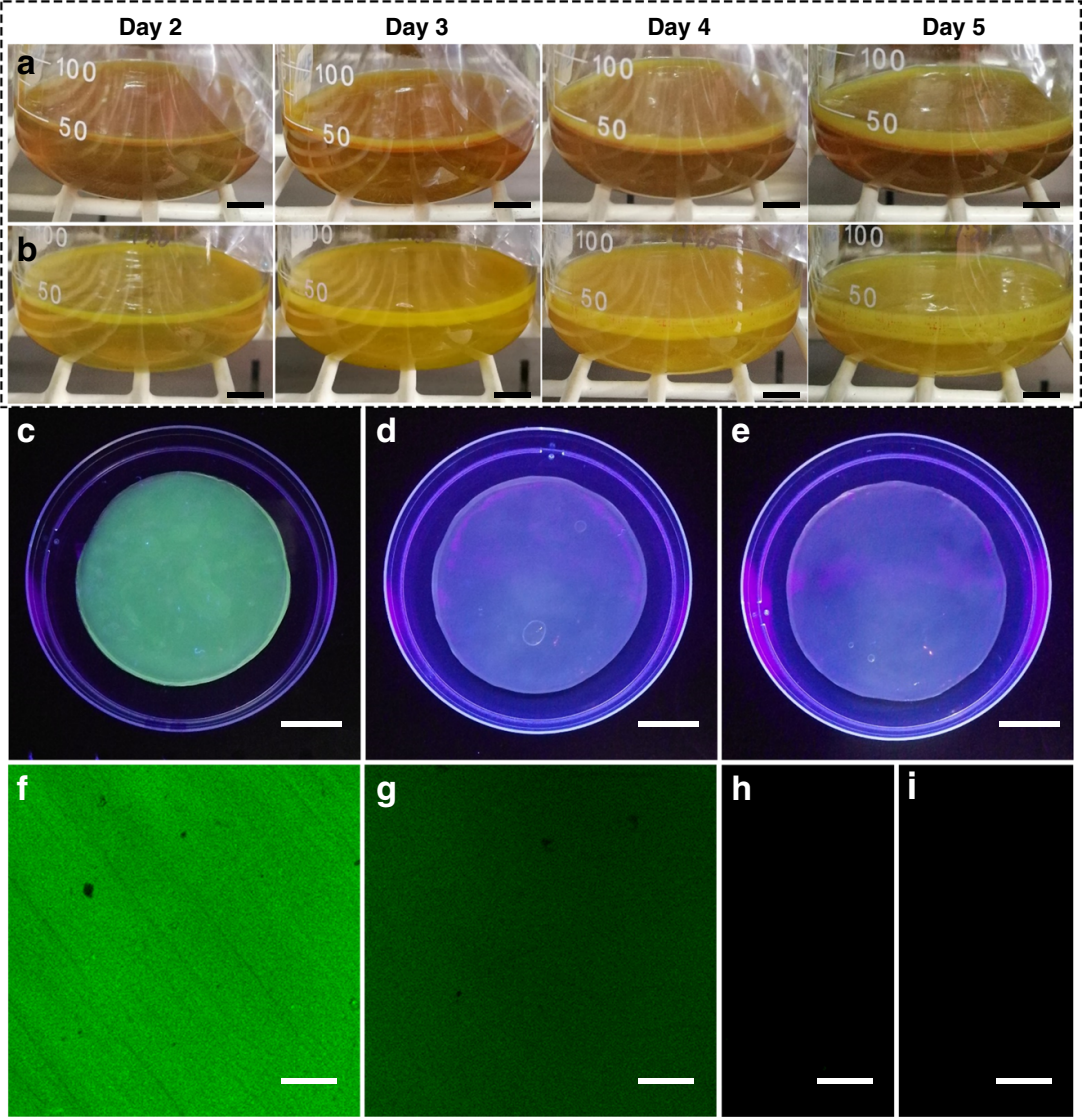
Biological incorporation of fluorescent-tagged glucose (6CF-Glc) into BC fibers. **a, b** Images of 6CF-BC fermentation under standard conditions (30 °C) on days 2, 3, 4, and 5. The thickness of **a** HC-6CF-BC (high concentration of 6CF-Glc, 0.95 mg mL^−1^) was thinner than **b** LC-6CF-BC (low concentration of 6CF-Glc, 0.38 mg mL^−1^). The scale bar corresponds to 1 cm. **c**–**e** Images of 6CF-BC, BC, and Im-6CF/BC observed under UV light (365 nm). **c** 6CF-BC showed green fluorescence, whereas **d** Im-6CF/BC and **e** BC showed no fluorescence. The scale bar corresponds to 2 cm. **f**–**i** Images of 6CF-BC, BC, and Im-6CF/BC observed with CLSM (excitation wavelength 488 nm). **f** HC-6CF-BC and **g** LC-6CF-BC showed green fluorescence, whereas **h** Im-6CF/BC and **i** BC showed no fluorescence. The scale bar corresponds to 100 μm

6CF-BC was obtained by adding 6CF-Glc to the culture medium of *K. sucrofermentans*. Based on the feeding ratios of 6CF-Glc, the obtained 6CF-BC was divided into two groups, HC-6CF-BC (high concentration of 6CF-Glc, 0.95 mg mL^−1^) and LC-6CF-BC (low concentration of 6CF-Glc, 0.38 mg mL^−1^). For contrast, the neat BC was immersed in a sterile fermentation medium containing 6CF-Glc and allowed physical adsorption (Im-6CF/BC). The synthesis images of 6CF-BC with different concentrations of 6CF-Glc from days 2 to 5 are shown in Fig. 2a,b. The thickness of HC-6CF-BC (Fig. 2a) was thinner than LC-6CF-BC (Fig. 2b), indicating that the structure of BC was affected by the increasing concentration of 6CF-Glc. We proposed that 6CF-Glc impeded the formation of intermolecular hydrogen bonds between cellulose and water molecules.

**Fig. 3:**
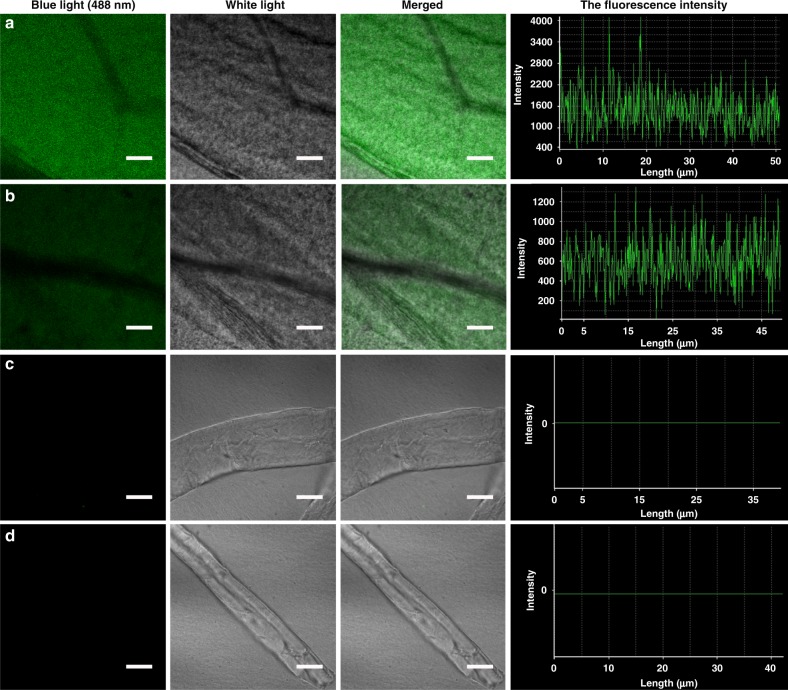
Detection of fluorescence intensity. The fluorescence intensity of HC-6CF-BC, LC-6CF-BC, Im-6CF/BC and BC was detected using CLSM under the same parameter settings. **a** HC-6CF-BC; **b** LC-6CF-BC; **c** Im-6CF/BC; **d** BC. The average fluorescence intensities of **a** HC-6CF-BC and **b** LC-6CF-BC were 1492 a.u. and 612 a.u., respectively. However, no fluorescence was observed in **c** Im-6CF/BC and **d** BC, and both fluorescence intensities were zero. The scale bar corresponds to 10 μm

To remove the residual medium and microorganisms, the samples, including BC, 6CF-BC and Im-6CF/BC, were treated with NaOH (2%, w/v) and then observed under UV light and with CLSM. Green fluorescence was observed on 6CF-BC samples (Fig. 2c, f, g), and no fluorescence was found for Im-6CF/BC and BC (Fig. 2d, e, h, i). The results showed that 6CF-Glc was integrated into BC by the metabolism of *K. sucrofermentans* and further eliminated interference from the fluorescence of 6CF-BC caused by surface contamination. In addition, the fluorescence distribution of HC-6CF-BC was more uniform than that of Ch-6CF/BC (Supplementary Figure 1). The fluorescent signal of HC-6CF-BC was stronger than that of LC-6CF-BC when observed with CLSM and fluorescence microscopy (Fig. 3 and Supplementary Figure 6), demonstrating that the fluorescence intensity of functional BC was controlled by adjusting the concentration of 6CF-Glc in the culture medium.

### Structural characterization of functional BC

**Fig. 4:**
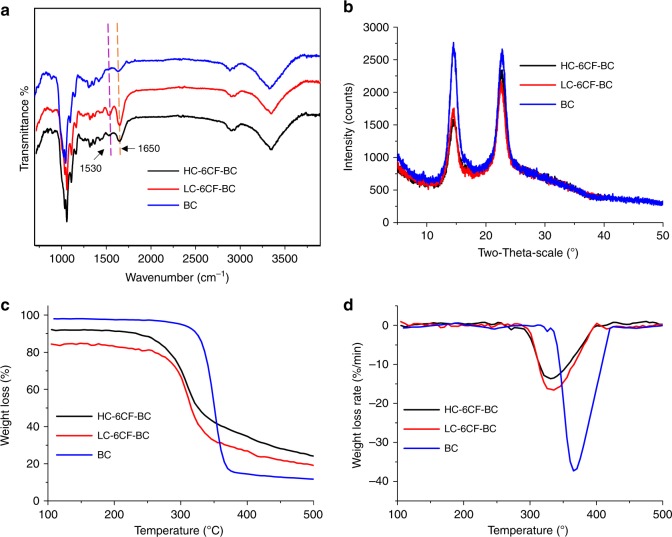
Structural characterization of different BC samples. **a** FT-IR ATR spectra of HC-6CF-BC (black), LC-6CF-BC (red) and BC (blue), showing a structural modification. New peaks appeared at 1530 cm^−1^ and 1650 cm^−1^ in the spectra of HC-6CF-BC and LC-6CF-BC, demonstrating the successful introduction of 6CF into BC. **b** XRD spectra of HC-6CF-BC (black), LC-6CF-BC (red) and BC (blue), showing changes in crystallinity. **c** TG and **d** DTG analysis of HC-6CF-BC (black), LC-6CF-BC (red) and BC (blue). The lowest endothermic peak of HC-6CF-BC (black) and LC-6CF-BC (red) was 330 °C and 335 °C, respectively, and the peak was 365 °C for BC (blue). The crystallization temperature of 6CF-BC was lower than BC by 30–35 °C, indicating an increase in the amorphous cellulose content in 6CF-BC

FT-IR ATR spectroscopy was performed to identify the modification of BC. Compared with BC, new peaks appeared at 1530 cm^−1^ (C=O stretching), 1650 cm^−1^ (C=O stretching in CO-NH), and 1453 cm^−1^ (benzene ring vibration) in the spectra of 6CF-BC (Fig. 4a, black and red). Furthermore, a wide absorption band appeared at 3354 cm^−1^ (OH stretching). The peak at 2904 cm^−1^ (CH_2_ anti-symmetric stretching), an inconspicuous peak at 2853 cm^−1^ (CH_2_ symmetric stretching), a peak at 1163 cm^−1^ (anti-symmetric stretching of C-O-C) and a peak at 896 cm^−1^ (β-1,4-glycosidic bonds) became sharper^30,32,33^. The shift of peaks to lower wavenumber regions was mainly caused by the abundant inter and intramolecular hydrogen bonds in BC^34^. Acylamino was the characteristic group representing the combination of 6CF and glucose. Therefore, the new peaks appeared at 1530 cm^−1^ and 1650 cm^−1^, verifying the successful introduction of 6CF into BC.

The pattern of X-ray diffraction (XRD) (Fig. 4b) showed that all samples had two absorption peaks, appearing at 14.5° and 22.6°, corresponding to the (110) and (200) planes of the cellulose form I-β crystal^35,36^. These results revealed that the introduction of 6CF had no significant effect on the crystal structure of BC, indicating that 6CF modification may mainly occur in the amorphous domains of BC. However, the crystallinity of HC-6CF-BC (71.8%) and LC-6CF-BC (90.0%) was lower than that of BC (98.3%), demonstrating that 6CF-Glc interfered with the orderly arrangement of BC chains and reduced the crystallinity. Therefore, the crystallinity was decreased with the increasing concentration of 6CF-Glc.

Thermogravimetric (TG) analysis (Fig. 4c) showed that thermal decomposition began at 230 °C for both HC-6CF-BC (black) and LC-6CF-BC (red), and 300 °C for BC (blue). The differential thermal gravity (DTG) (Fig. 4d) showed that the lowest endothermic peaks of HC-6CF-BC (black) and LC-6CF-BC (red) were 330 °C and 335 °C, respectively, and it was 365 °C for BC (blue). The maximum weight loss rate occurred at this time, corresponding to pyrolysis of the β-1,4-glycosidic bond. The endothermic peak of 6CF-BC was wider (from 220 °C to 450 °C) and less intense, whereas it was sharper (from 280 °C to 390 °C) and stronger in the spectrum of BC. The crystallization temperature of 6CF-BC was lower than that of BC by 30–35 °C (Fig. 4d), indicating that the thermal stability of 6CF-BC was poorer than that of BC and that the amorphous cellulose content was increased in 6CF-BC^37^. These results were consistent with the results of XRD.

**Fig. 5:**
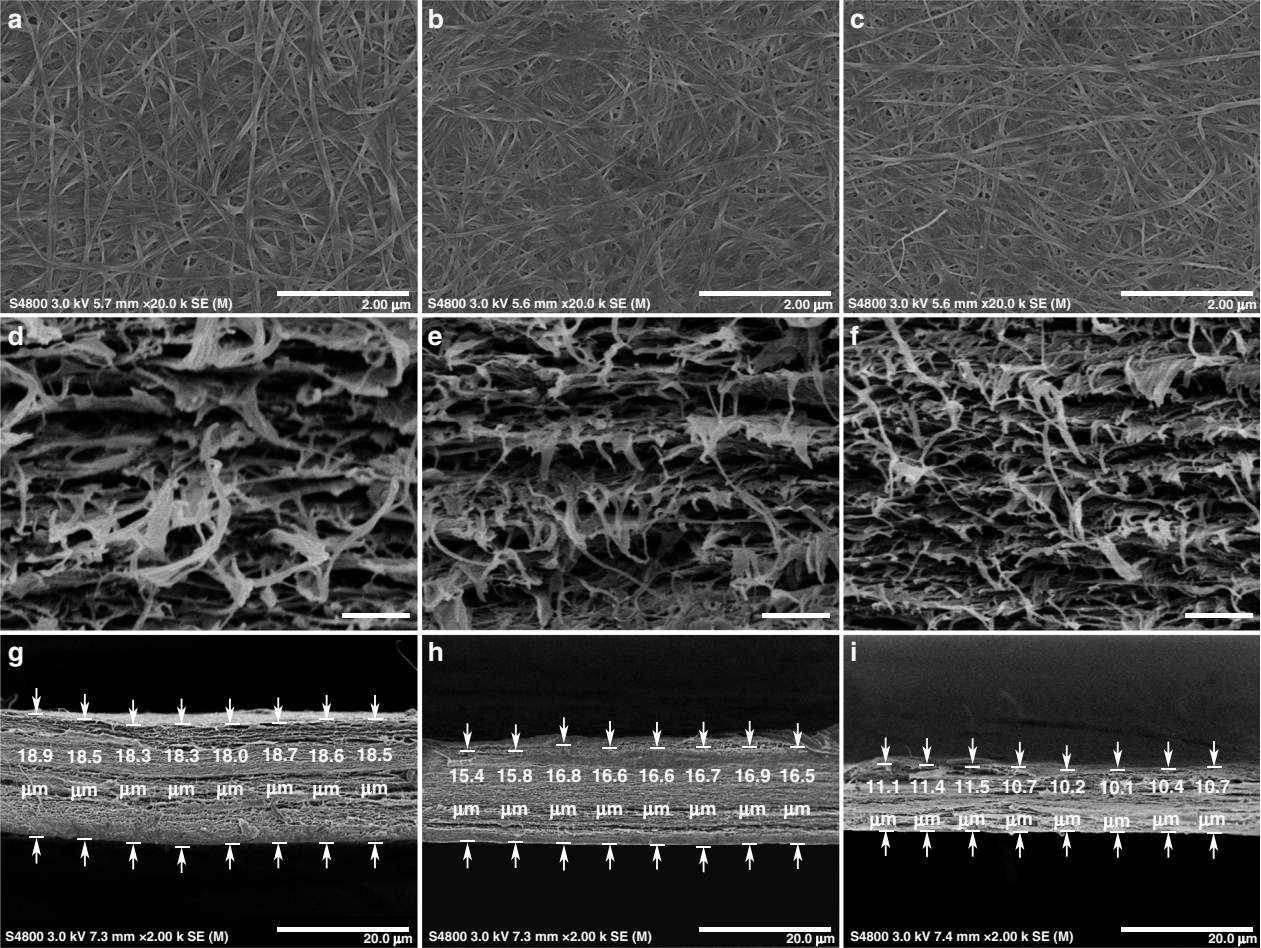
Microstructure observation of BC through SEM. **a**–**c** Top view of different BC samples. The diameters of different BC fibers showed no significant differences, whereas the pore sizes of **a** HC-6CF-BC and **b** LC-6CF-BC were slightly larger than that of **c** BC. The scale bar corresponds to 2 μm. **d**–**i** Cross-section of different BC samples. The gaps of **d** HC-6CF-BC were significantly larger than those of **e** LC-6CF-BC and **f** BC samples. Moreover, **g** HC-6CF-BC was also thicker than **h** LC-6CF-BC and **i** BC samples. **d**–**f**, The scale bar corresponds to 400 nm. **g**–**i**, The scale bar corresponds to 20 μm

Scanning electron micrographs (SEM) showed that there was no significant differences in the diameters of BC fibers for HC-6CF-BC (Fig. 5a), LC-6CF-BC (Fig. 5b), and BC (Fig. 5c). However, the pore size of BC was enlarged with the increasing concentration of 6CF-Glc. We hypothesized that 6CF-Glc interfered with the arrangement of BC chains and reduced the interactions of hydrogen bonds among cellulose chains, leading to their loose arrangement and the enhancement of pore size. This suspicion was also confirmed through the cross-sectional SEM of different BC samples (Fig. 5d–f). The gaps of HC-6CF-BC were significantly larger than those of the BC samples. Moreover, the dried HC-6CF-BC was thicker than the dried LC-6CF-BC and dried BC (Fig. 5g–i). Therefore, 6CF may have a certain influence on the crystal structure and crystallinity of BC. The results were consistent with the XRD and TG analysis.

Finally, we performed tensile tests on BC, LC-6CF-BC,and HC-6CF-BC (Supplementary Figure 7 and Supplementary Table 1). The 6CF-BC had a lower elastic modulus and tensile strength than BC. Concomitantly, the values of elongation at break were enhanced with the increasing concentration of 6CF-Glc, and HC-6CF-BC showed the highest values. Based on the results of XRD, TG, and SEM, the crystallinity of 6CF-BC was reduced by adding 6CF-Glc, which further influenced the mechanical properties of 6CF-BC.

## Discussion

This study micro-biosynthesizes functional BC successfully. Traditional modifications on BC are mainly conducted with fermentation products using chemical modifications or physical coatings. Traditional physical modification has primarily concentrated on the sputter coating of metal oxide or nanoparticles and evaporation-induced self-assembly based on cellulose nanocrystals^24,38^, which needed complex operations and was not suitable for large-scale production. Due to the poor solubility of BC, chemical modification was often conducted with special solvents or in a heterogeneous reaction system based on cellulose nanocrystals, which have several drawbacks, such as low efficiency, waste of raw materials and an increase in the burden of waste discharge treatment^29^. Our work aims to circumvent these limitations. A recent study reported a biosynthesis system that can be utilized to produce functional plant cellulose^30^. The key point is the modification of glucose, which is then used as the carrier to introduce functional moieties into plant cellulose. Glucose is a soluble polysaccharide, which is a carbon source for the synthesis of BC by *K. sucrofermentans*. Therefore, we anticipate that utilizing the functionalized glucose as the carbon source can help produce BC with tailored properties using a microbial fermentation system.

We modified the glucose with 6CF, and the product (6CF-Glc) was added to the fermentation medium of *K. sucrofermentans* to synthesize functional 6CF-BC. We observed that 6CF-BC had a green fluorescence based on UV and CLSM detection. We further modified BC using a chemical method. Ch-6CF/BC showed green fluorescence, but the fluorescence signal distribution was less uniform than that of 6CF-BC, which may be related to the heterogeneous reaction system. Unexpectedly, the FT-IR ATR spectrum of Ch-6CF/BC was consistent with that of BC, and no characteristic absorption peaks appeared at 1740 cm^−1^ (C=O stretching in ester). This result revealed that no ester bonds formed on Ch-6CF/BC. Therefore, the method^21^ we referred to was not suitable for the chemical modification of BC with 6CF. More importantly, due to the poor solubility of 6CF in water, the chemical modification to BC required a large amount of organic solvent (DMF), which not only increased the cost but also caused environmental pollution due to its toxic side effects^39^. Thus, the biological modification method is more suitable than the chemical method for large-scale production. Therefore, the biosynthesis system was more efficient than chemical modification, particularly when modified with water-insoluble materials.

We then tested the structural characteristics of 6CF-BC, Ch-6CF/BC, and BC. 6CF-BC was synthesized by microbial metabolism but not through the physical adsorption of 6CF-Glc, which was verified by FT-IR ATR. According to the results of XRD, TG, and SEM, the microstructure of 6CF-BC fibers was maintained and remained type I-β cellulose compared to BC. However, due to the addition of 6CF, the regular arrangement among the glucose molecules was disrupted, which in turn reduced the crystallinity and thermal stability of 6CF-BC. Furthermore, with an increase in 6CF-Glc concentration, the tensile strength was also reduced while the strain was increased, which was consistent with the results in a previous study^30^.

**Fig. 6:**
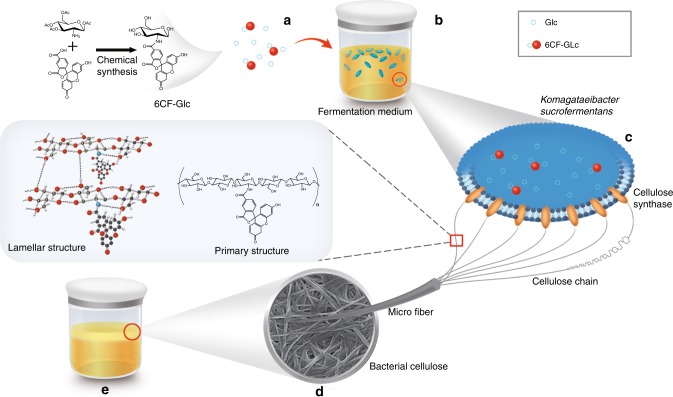
Synthesis of 6CF-BC based on an in situ microbial fermentation method. Glucose (Glc) was modified with 6CF, and the 6CF-Glc was used as a carbon source for *K. sucrofermentans* fermentation to obtain 6CF-BC through microbial metabolic pathways. **a** Glc and 6CF-Glc molecules. **b** Microorganism fermentation. **c** The synthesis of 6CF-BC fibers through *K. sucrofermentans*. **d** Microstructure of 6CF-BC. **e** The 6CF-BC pellicle obtained through microorganism fermentation

The C1 position is essential for the polymerization of glucose units into fibers^30^. 6CF was attached to the C2 position of glucose, leaving the C1 and C4 positions free for cellulose formation. Thus, 6CF-Glc could be utilized by *K. sucrofermentans*. Based on the metabolic pathway of glucose^40^, the metabolism of 6CF-Glc was proposed as followed. After 6CF-Glc entered *K. sucrofermentans*, it might be first phosphorylated by glucokinase to obtain 6CF-Glc-6-phosphate, which might be further converted to 6CF-Glc-1-phosphate by the isomerization of glucose phosphate isomerase. Then, glucose pyrophosphorylase might convert the 6CF-Glc-1-phosphate to uridine diphosphate-6CF-Glc (UDP-6CF-Glc). Finally, UDP-6CF-Glc might be ligated via β-1,4-glycosidic linkage to synthesize 6CF-BC (Fig. 6). Though the hypothesis of 6CF-Glc metabolism was proposed base on the metabolic pathway of glucose, it remained to be further authenticated.

Overall, our results verified the notion that BC could be functionalized by using a microbial synthesis system. This method can be extended to the production of other functional materials. The key technologies for this method are (i) the design of metabolic substrates (functionalized carbon and nitrogen sources) and (ii) an understanding of the mechanism of substrate-enzyme recognition and substrate resistance. Based on this premise, the yield can also be improved, and the varieties of metabolized substrates can be expanded. For example, Thongsomboon et al. reported that functionalized cellulose can be naturally produced by modifying the genetically engineered strain^41^. The optimal functionalized method should be (i) green and environmentally friendly, (ii) highly efficient, (iii) low cost, (iv) suitable for large-scale production, and (v) easily modified on specific sites. Therefore, there is a worldwide demand for environmental-friendly and sustainable production of new added-value chemicals and other materials based on biosynthesis methods^42,43^. Functional molecules can be efficiently assembled by utilizing the enzyme catalysis of microbial cell factories, breaking the bottlenecks of physical and chemical synthetic methods and producing functional materials and intermediate chemicals, particularly for those obtained with difficulty by using traditional methods. Therefore, although our data has several limitations, this method meets all five criteria, lending experimental support to our idea of an innovative biosynthesis method to functionalize BC. Our work not only confirmed that microorganisms can metabolize and utilize the modified glucose (6CF-Glc) but also provided a method for the in situ synthesis of functional materials and possibly functional drugs in other microbial synthetic systems.

## Methods

### Strains and culture medium

*K. sucrofermentans* was used for BC fermentation. H-S basic medium (g L^−1^) consisted of glucose 25, yeast extract 5, peptone 5, citric acid monohydrate 1.2 and Na_2_HPO_4_ 2.7. The volume of all fermentation media was constant (40 mL), and they were sterilized at 115 °C for 30 min.

### Synthesis of 6CF-Glc

The synthesis method was based on a previous study^30^, with slight modifications. Dried N,N-dimethylformamide (DMF, 3 mL) was used as solvent for synthesis of the 6CF-Glc conjugate to dissolve 50 mg of 6CF (AAT Bioquest, Inc., USA). Subsequently, 20 mg of N,N,N′,N′-tetramethyl-O-(N-succinimidyl) uronium tetrafluoroborate (Macklin Biochemical Co., Ltd., Shanghai, China) and 100 µL of N,N-diisopropylethylamine (DIPEA, Macklin Biochemical Co., Ltd., Shanghai, China) were added and incubated for 30 min at room temperature under inert conditions to activate 6CF. Dried DMF with DIPEA (100 µL) was used to dissolve 25 mg of 1, 3, 4, 6-tetra-O-acetyl-2-amino-2-deoxy-β-D-glucopyranose hydrochloride (Aladdin, Shanghai, China) as parallel-group. The 1,3,4,6-tetra-O-acetyl-2-amino-2-deoxy-β-D-glucopyranose solution was mixed with activated 6CF solution and incubated overnight at room temperature under inert and dark conditions. The product was an orange solid. Then, column chromatography (silica) and thin-layer chromatography (dichloromethane/MeOH 10:1) were applied to remove excess coupling reagents and elute the product, respectively. A solution of NaOH (3 mL, 0.01 M) was added to 15 mg of the orange conjugate for deacetylation. The solution was left to react for 10 min at room temperature after ultrasonicated for 5 s. Thin-layer chromatography (dichloromethane/MeOH 10:1) was used to monitor the extent of deacetylation. HCl (3 mL, 0.01 M) was applied to stop the reaction by neutralizing the mixture to pH 7.0. At last, the target product was obtained by vacuum drying and characterized by UV-Vis spectroscopy.

### Microbial fermentation

*K. sucrofermentans* was inoculated in 7% (v/v) H-S basic medium and cultured at 30 °C for 5 days. The obtained BC samples were treated with sodium hydroxide solution (2%, w/v) overnight at 60 °C, and then washed thoroughly with deionized water. All samples were dried at 60 °C in a constant temperature drying oven.

### The selectivity of 6CF on *K. sucrofermentans*

*K. sucrofermentans* was cultured with H-S basic medium supplemented with 6CF (50 mg) at 30 °C for 5 days. Then, the obtained BC was washed thoroughly with deionized water.

### Preparation of 6CF-BC

*K. sucrofermentans* was cultured with H-S basic medium supplemented with 6CF-Glc (0.38 mg mL^−1^ or 0.95 mg mL^−1^) at 30 °C for 5 days. Then, 6CF-BC was treated with sodium hydroxide solution (2%, w/v) overnight at 60 °C and then washed with deionized water thoroughly until no fluorescence was detected in the residual water. All samples were dried at 60 °C in a constant temperature drying oven.

### Preparation of Ch-6CF/BC

The preparation method was based on a previous study^29^, with slight modifications. BC was produced by microbial fermentation under standard conditions mentioned above without 6CF-Glc. N,N-dimethylacetamide (DMF) was selected as the solvent for 6CF because of its poor solubility in water. 6CF (50 mg) was mixed with dried DMF (20 mL), N,N,N′,N′-tetramethyl -O-(N-succinimidyl) uronium tetrafluoroborate (20 mg) and DIPEA (100 µL) and left to activate for 30 min at room temperature under inert conditions. Then, neat BC was soaked in activated 6CF DMF solution for 30 min at room temperature under inert conditions and dried at 80 °C. Next, the chemically modified BC (Ch-6CF/BC) was washed with excess DMF and dried at room temperature.

### Preparation of Im-6CF/BC

Neat BC was soaked in the H-S basic medium supplemented with 6CF-Glc (0.95 mg mL^−1^) at 30 °C and allowed to physically adsorb for 5 days. Then, the obtained BC (Im-6CF/BC) was treated with sodium hydroxide solution (2%, w/v) overnight at 60 °C and washed with deionized water until no fluorescence was detected in the residual water. All samples were dried at 60 °C in a constant temperature drying oven.

### CLSM

CLSM (FluoView FV1000, Olympus, Japan) was used for observing different BC fibers. Images were recorded at 488 nm (excitation) and 515 nm (emission).

### FT-IR ATR

FT-IR spectroscopic measurements of glucose, 6CF-Glc, BC, Im-6CF/BC, and 6CF-BC were recorded on a Nicolet 6700 FTIR Spectrometer (Thermo Fisher, USA) at 25 °C and 30% relative humidity. All spectra were recorded in the spectral range between 700 and 4000 cm^−1^ with a resolution of 4 cm^−1^, averaging 32 scans. The experiment was repeated at least three times.

### ^1^H NMR

^1^H NMR spectra were recorded using the AVANCE-III 600 NMR spectrometer (600 MHz, Bruker BioSpin, Switzerland). 6CF-Glc was dissolved in CD_3_OD. Data were processed using MestRENova (v.12.0.2, MestRElab Research, Spain).

### Liquid chromatography–mass spectrometry (LC-MS)

UHPLC analysis was carried out using an Ultimate 3000 UHPLC (Thermo, USA) with a Thermo Acclaim RSLC C18 column (2.1 mm × 100 mm, 2.2 μm), and Thermo online UHPLC filter (2.1 mm, 0.2 μm) was used for the chromatographic separation. The mobile phase A consisted of 0.1% of formic acid in water, and the mobile phase B was composed of CH_3_CN with 0.1% formic acid. The elution gradient was started at 2% B for the first 2 min with flow rate of 0.2 mL min^−1^, stepping to 40% B at 6 min, holding at 40% B until 10 min, and returning to 2% B at 10.1 min, holding these conditions at 15 min and stopping the controller. The injection volume was 5 μL and the temperature of column was 30 °C.

A Compact Q-TOF mass spectrometry (Bruker Daltonics, Billerica, USA) with an ESI source in negative ion mode with HyStar 3.2 software (Bruker Daltonics, Billerica, USA) was used to link the LC and the MS, using the following operation parameters: capillary voltage 3800 V, dry temperature 200 °C, nebulizing gas of 1.5 bar, drying gas (N_2_, purity 99.99%) flowing of 4.5 L min^−1^. High-resolution MS and MS/MS spectra were acquired in the range 50–1300 m/z. The collision gas was high purity nitrogen (99.99%). The data were collected by auto MS/MS acquisition with a MS scan rate of 1 spectra s^−1^ and MS/MS scan rate of three precursor was acquired per cycle, active exclusion after 3 spectra and 1.0 min. Otof Control software (Bruker Daltonics, Billerica, USA) was used to carry out mass spectrometer control and data acquisition, and Compass Data Analysis software (Bruker Daltonics, Billerica, USA) was applied for data analysis.

### XRD

The processed dried sample was analyzed on a D8 Advance diffractometer (Bruker, Germany) using Cu Kα radiation (λ = 0.154 nm) at 40 kV and 40 mA with a scanning range of 5–50°. The experiment was repeated at least three times.

### TG analysis

The measurement was performed on a thermogravimetry analyzer (STA449F5 Jupiter, Netzsch, Germany). The samples (2–3 mg) were weighed accurately into aluminum pans and sealed. The aluminum pans were scanned from 25 °C to 600 °C with heating rates of 10 °C min^−1^ under a N_2_ atmosphere (50 mL min^−1^). For the buoyancy correction, a baseline was recorded using approximately 40 mg of dry Al_2_O_3_ and subtracted from the sample measurements. The data were analyzed using Origin Lab 8 (Origin Lab Corporation, USA). The experiment was repeated at least three times.

### SEM observation

The morphologies of BC, 6CF-BC and Im-6CF/BC samples were observed using an S-4800 SEM (Hitachi, Japan). The samples were mounted onto an aluminum stub, coated by platinum sputter and analyzed using SEM with a 3.0 kV acceleration voltage.

### Tensile testing

The dried BC and 6CF-BC samples were cut into ribbons (1 cm × 10 cm). Each sample was fixed at both ends of the machine grips. An Instron universal tester 5966 (Instron, Norwood, MA, USA) was used to apply unidirectional tension while recording the force and extension. The loading rate was maintained at 10 mm min^−1^. The tensile fracture stress was determined as the values obtained from the initial linear section of the stress-strain curves. The experiment was repeated at least six times.

### Statistics

Dots in figures report values obtained from independent replicates unless otherwise noted. Data in tables are expressed as means ± s.d. Statistical analyses were performed using SPSS Statistics 21.0 (IBM, New York, USA). Two-tailed Student *t*-tests were used in all comparisons. *P* < 0.05 was considered statistically significant throughout the study.

## Supplementary information

Supplementary Information

Reporting Summary

## Data Availability

The data that support the findings of this study are available from the corresponding author upon reasonable request.
