# Supplementary material for: A natural in situ fabrication method of functional bacterial cellulose using a microorganism
Source: Nat Commun. 2019 Jan 25;10:437. doi: 10.1038/s41467-018-07879-3 (PMC6347598; doi:10.1038/s41467-018-07879-3)
Supplement: Supplementary file 1 — Supplementary Information [file 41467_2018_7879_MOESM1_ESM.pdf]

Supplementary Information

A natural *in situ* fabrication method of functional bacterial cellulose using a microorganism

Gao et al

Supplementary Figure 1

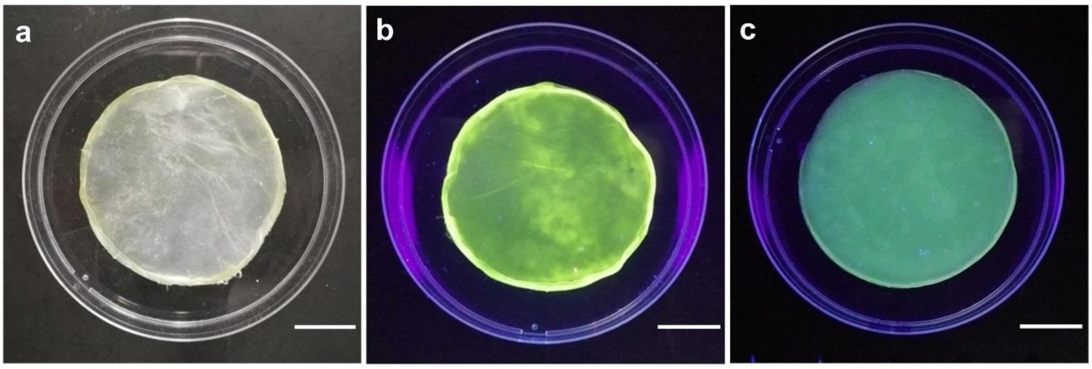

Supplementary Figure 1 Images of Ch-6CF/BC and HC-6CF-BC samples. **a**, The image of Ch-6CF/BC observed under white light. **b**-**c**, The images of different BC samples observed under ultraviolet light (365 nm). Green fluorescence was observed on both (**b**) Ch-6CF/BC and (**c**) HC-6CF-BC, whereas the fluorescence distribution of HC-6CF-BC was more uniform than that of Ch-6CF/BC. The scale bar corresponds to 2 cm.

Supplementary Figure 2

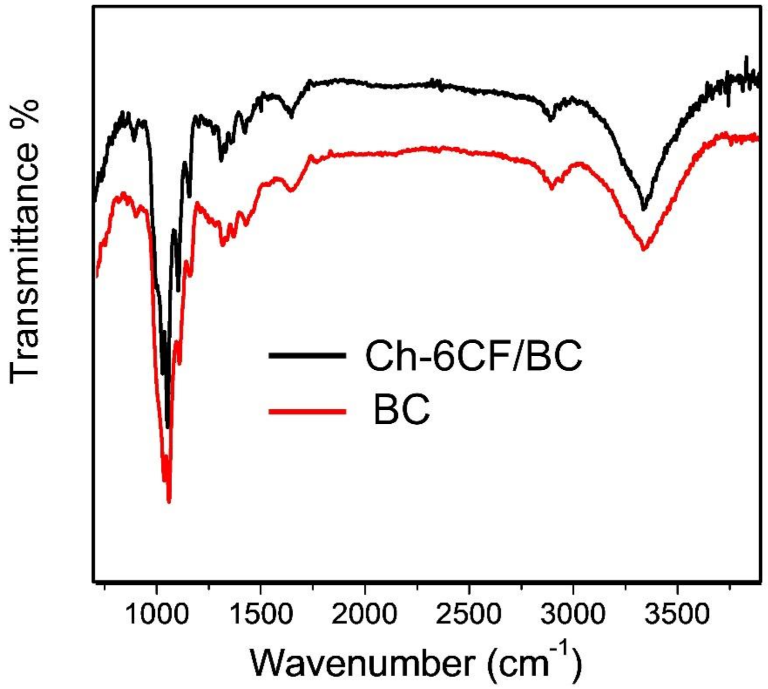

Supplementary Figure 2 FT-IR ATR spectra of Ch-6CF/BC and BC. The spectra showed that the absorption peaks of Ch-6CF/BC (black) were the same as those of BC (red), and no characteristic absorption peaks appeared at 1740 cm^{-1} (C=O stretching in ester). The result revealed that no ester bonds were formed on Ch-6CF/BC, indicating that 6CF was physically adsorbed on the BC.

Supplementary Figure 3

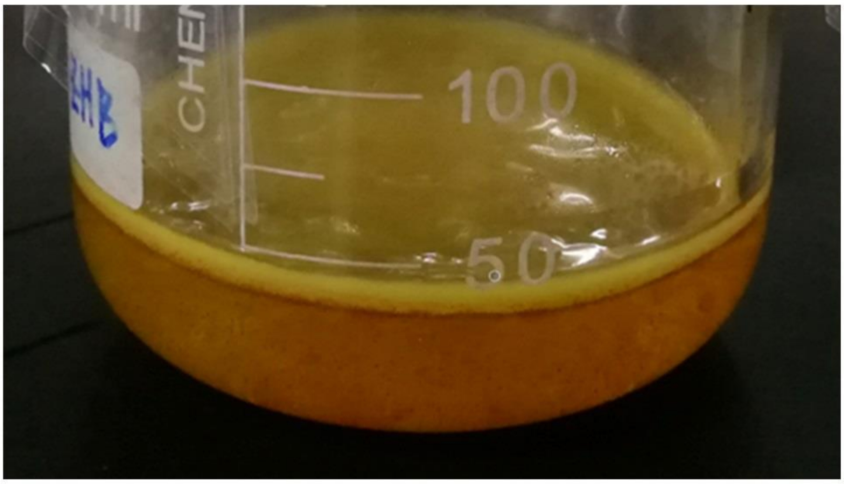

Supplementary Figure 3 A photo of BC sample produced by *K. sucrofermentans* cultivated in H-S medium supplemented with 6CF. *K. sucrofermentans* was cultivated in H-S fermentation medium supplemented with 6CF (50 mg) under standard conditions (30ºC, 5 days). A complete BC film was obtained on the surface of the culture medium, indicating that 6CF had no clear impact on the growth and metabolism of *K. sucrofermentans*.

Supplementary Figure 4

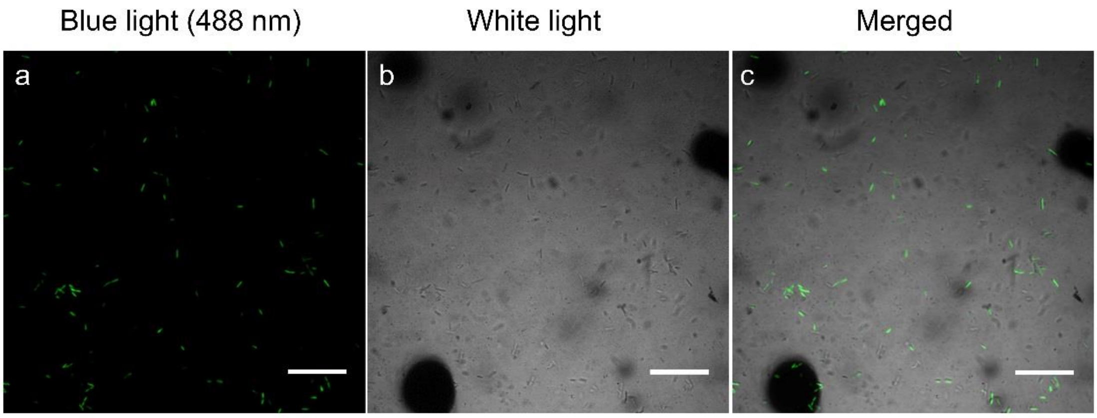

Supplementary Figure 4 CLSM observation. CLSM images (emission wavelength, 488 nm) of BC samples produced by cultivating *K. sucrofermentans* in H-S fermentation medium supplemented with 6CF under standard conditions. **a**, Blue light, 488 nm. **b**, White light. **c**, Merged. Green fluorescence was observed on a part of *K. sucrofermentans*, demonstrating that 6CF could enter the cell body of *K. sucrofermentans*. The scale bar corresponds to 20 μm.

Supplementary Figure 5

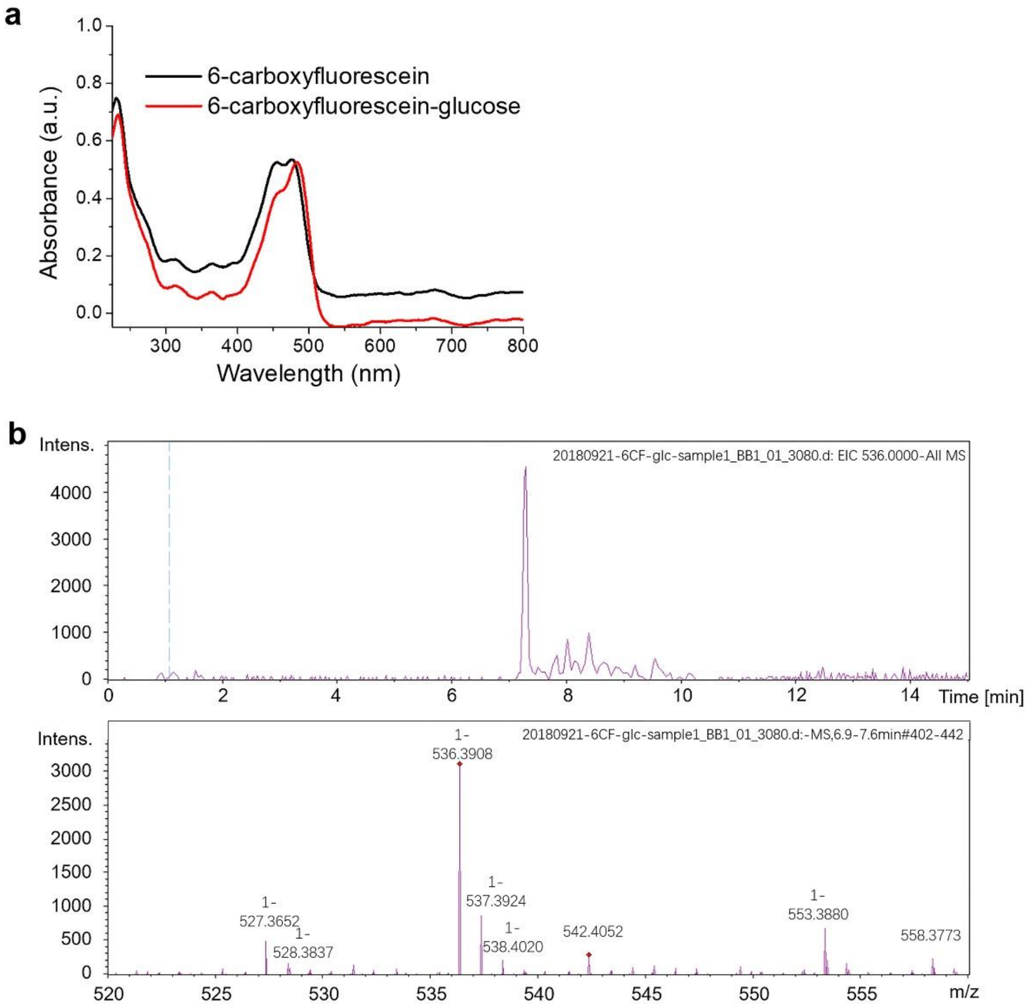

Supplementary Figure 5 Characteristic analysis of 6CF and 6CF-Glc. **a**, UV-Vis spectra measured in distilled water, pH 6. Black line: 6-carboxyfluorescein with bands at 231/455/477 nm. Red line: 6CF-Glc with bands at 232/457/481 nm. The observed peak with a slight redshift demonstrated the conjugation of 6-carboxyfluorescein to glucose. **b**, LC-ESI-MS spectra (m/z, CH_3CN/formic acid 0.1%): found 536.39 $[M-H]^-$. ^1H NMR (600 MHz, CD_3OD) δ (ppm): 8.15 (dd, *J* = 8.04, 1.68 Hz, 1H), 8.01 (d, *J* = 8.04, 1H), 7.80 (d, *J* = 1.56, 1H), 7.09 (d, *J* = 9.18, 2H), 6.55-6.61 (m, 5H), 6.16 (dd, *J* = 17.40, 10.20 Hz, 1H), 6.04 (dd, *J* = 17.40, 2.34 Hz, 1H), 5.50 (dd, *J* = 10.20, 2.28 Hz, 1H), 3.55 (m, 1H), 3.18 (m, 2H).

Supplementary Figure 6

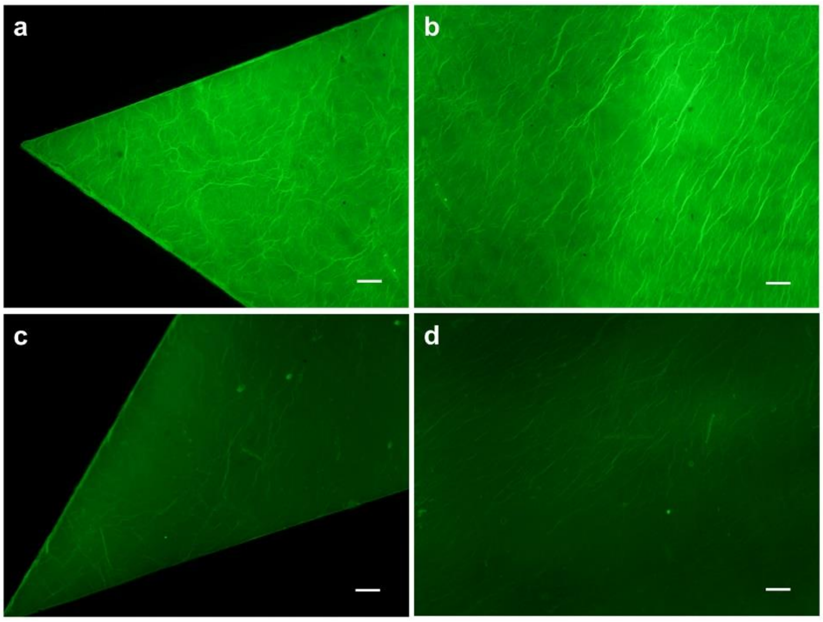

Supplementary Figure 6 Fluorescence observation of HC-6CF-BC and LC-6CF-BC with a fluorescence microscopy. **a**-**b**, represent two different fields of view of HC-6CF-BC. **c**-**d**, represent two different fields of view of LC-6CF-BC. The fluorescent signal of (**a**, **b**) HC-6CF-BC was stronger than that of (**c**, **d**) LC-6CF-BC. The scale bar corresponds to 200 μm.

Supplementary Figure 7

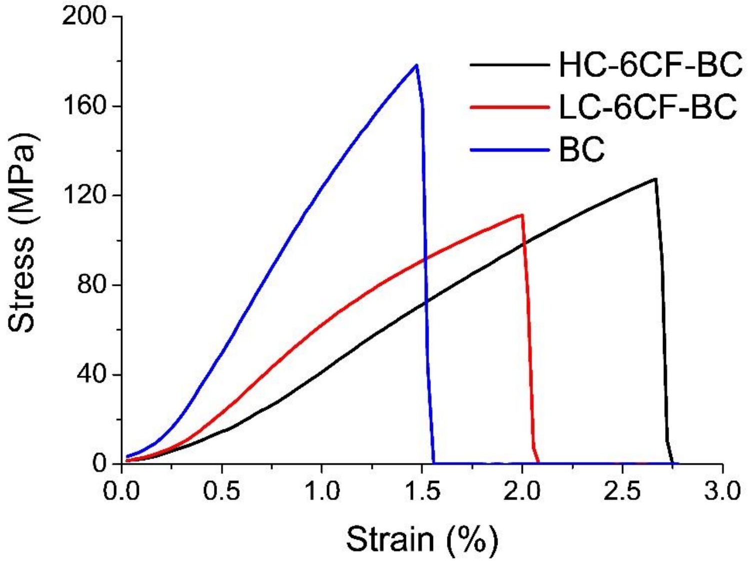

Supplementary Figure 7 Stress-strain graphs of different BC samples. The width and thickness of samples was measured using a Vernier caliper and the cross-sectional SEM images of samples (Fig. 5g-i). The 6CF-BC (black and red) had a lower elastic modulus and tensile strength than BC (blue). Concomitantly, HC-6CF-BC showed the highest values of elongation at the break. The results demonstrated that the introduction of 6CF influenced the mechanical properties of BC.

Supplementary Table 1

Sample	Elastic Modulus (MPa)	Elongation at break (%)	Tensile strength (MPa)
HC-6CF-BC	9315.10 ± 315.22	2.77 ± 0.21	134.56 ± 21.65
LC-6CF-BC	8183.62 ± 445.36	2.08 ± 0.35	115.94 ± 35.87
BC	13409.94 ± 558.77	1.57 ± 0.48	189.89 ± 29.55

Supplementary Table 1 Mechanical properties of different BC samples. For each sample, at least 6 individual samples were measured to calculate a mean value. Data are expressed as means ± s.d.
